# Adult T-Cell Leukemia: A Review of Epidemiological Evidence

**DOI:** 10.3389/fmicb.2012.00322

**Published:** 2012-09-10

**Authors:** Masako Iwanaga, Toshiki Watanabe, Kazunari Yamaguchi

**Affiliations:** ^1^Graduate School of Public Health, Teikyo UniversityTokyo, Japan; ^2^Graduate School of Frontier Sciences, The University of TokyoTokyo, Japan; ^3^Department of Safety Research on Blood and Biologics, National Institute of Infectious DiseasesTokyo, Japan

**Keywords:** adult T-cell leukemia, ATL, epidemiology, human T-cell leukemia virus type I, HTLV-1

## Abstract

Adult T-cell leukemia (ATL) is an aggressive T-cell malignancy caused by human T-cell leukemia virus type I (HTLV-1) infection and often occurs in HTLV-1-endemic areas, such as southwestern Japan, the Caribbean islands, Central and South America, Intertropical Africa, and Middle East. To date, many epidemiological studies have been conducted to investigate the incidence of ATL among general population or HTLV-1 carriers and to identify a variety of laboratory, molecular, and host-specific markers to be possible predictive factors for developing ATL because HTLV-1 infection alone is not sufficient to develop ATL. This literature review focuses on the epidemiology of ATL and the risk factors for the development of ATL from HTLV-1 carriers, while keeping information on the epidemiology of HTLV-1 to a minimum. The main lines of epidemiological evidence are: (1) ATL occurs mostly in adults, at least 20–30 years after the HTLV-1 infection, (2) age at onset differs across geographic areas: the average age in the Central and South America (around 40 years old) is younger than that in Japan (around 60 years old), (3) ATL occurs in those infected in childhood, but seldom occurs in those infected in adulthood, (4) male carriers have about a three- to fivefold higher risk of developing ATL than female, (5) the estimated lifetime risk of developing ATL in HTLV-1 carriers is 6–7% for men and 2–3% for women in Japan, (6) a low anti-Tax reactivity, a high soluble interleukin-2 receptor level, a high anti-HTLV-1 titer, and high levels of circulating abnormal lymphocytes and white blood cell count are accepted risk factors for the development of ATL, and (7) a higher proviral load (more than 4 copies/100 peripheral blood mononuclear cells) is an independent risk factor for progression of ATL. Nevertheless, the current epidemiological evidence is insufficient to fully understand the oncogenesis of ATL. Further well-designed epidemiological studies are needed.

## Introduction

Adult T-cell leukemia (ATL) was first reported as a distinct clinical entity in Japan in 1977 (Takatsuki et al., 1977; Uchiyama et al., 1977). The clustering of patients in the southwestern part of Japan propelled Japanese investigators to the interest that the disease could be virally induced. Subsequently, human T-cell leukemia virus type I (HTLV-1) was discovered as the causative virus for ATL (Poiesz et al., 1980; Yoshida et al., 1982). The discoveries of ATL and HTLV-1 ushered in the development of virology, oncology, molecular biology, epidemiology, and other fields of medicine.

The etiological association of HTLV-1 with ATL was established on the basis of the following findings: (1) all patients with ATL have antibodies against HTLV-1 (Hinuma et al., 1981, 1982), (2) geographical areas of high incidence of ATL patients correspond closely with those of high incidence of HTLV-1 carriers (The T- and B-Cell Malignancy Study Group, 1985), (3) HTLV-1 immortalizes human CD4 T cells *in vitro* (Hattori et al., 1981), and (4) monoclonal integration of HTLV-1 proviral DNA was demonstrated in ATL cells (Yamaguchi et al., 1984). Subsequently, the Japanese Lymphoma Study Group proposed the first diagnostic criteria for ATL in 1991, and the disease was classified into four clinical subtypes; acute, lymphoma, chronic, and smoldering (Shimoyama, 1991).

ATL patients have been reported mainly from HTLV-1-endemic areas. The global geographical distribution of HTLV-1 seropositive individuals has been well documented (Proietti et al., 2005). Areas with seroprevalence of more than 2% are recognized as high endemic regions (Gessain, 1996). The main endemic areas are Japan, the Caribbean islands, Central and South America, Central and South Africa, a part of the Middle East and Melanesia, and Aboriginal regions in Australia (IARC, 1996). Moreover, regional clustering of virus positivity and high incidence of ATL has been detected even within the endemic areas. The prevalence of HTLV-1 carriers in Europe, North America, China, and Korea is low (Proietti et al., 2005).

This literature review focuses on the epidemiology of ATL and the risk factors for the development of ATL from HTLV-1 carriers with asymptomatic status, while keeping information on the epidemiology of HTLV-1 to a minimum. A variety of study designs and settings, e.g., case series, nation wide surveys, and regional population-based studies using cancer registries were reported to assess incidence, prevalence, and other epidemiological information on ATL from many countries, mostly from Japan. However, there have been few prospective cohort studies to assess reliable incidence rate of ATL. Readers should keep in mind that all epidemiological studies have individual limitations in the case accumulation and the population setting.

## Incidence and Prevalence

### Japan

In Japan, approximately one million individuals are carriers of HTLV-1 (Tajima, 1990; Satake et al., 2012). Both HTLV-1 and ATL have been shown to be endemic in southwest districts (Kyushu and Shikoku Islands; Tajima, 1990; Satake et al., 2012). Several epidemiological studies have been conducted to estimate annual incidence of ATL in HTLV-1 carriers or general population, but the exact annual incidence of ATL is still unclear. Most of the studies estimated the incidence of ATL just by merging the number of cases of ATL in one population to the number of people in another population such as demographic statistics, blood donors positive for HTLV-1, or an existing group of HTLV-1 carriers. Few prospective studies were conducted (Table 1).

**Table 1:** **Epidemiological studies of ATL in literatures**.

Study design	Reference	Country	Targeted population	Size of population	No. ATL cases	Incidence rate (IR)	Lifetime risk (estimated cumulative risk)
Population-based descriptive study	Kondo et al. (1989)	Japan	Inhabitants of Uwajima City (an endemic area in Japan)	Data from the Statistics Bureau in 1981	Data from a survey in 1981–1987	Annual IR:	NA
				M + F: 290,464	M: 46	3.9 per 100,000 population	
					F: 34	6.1 per 100,000 aged over 30	
						6.6 per 100,000 aged over 40	
			HTLV-1 carriers aged over 30 years	Data from HTLV-1 screening in 1981	Data from a survey in 1981–1987	Annual IR: (per 100,000 HTLV-1 carriers aged over 30 years)	(0–79 years):
				M: 4,522	M: 46	Total: 85.0 M: 145.3	M: 6.9%
				F: 8,801	F: 34	F: 55.2	F: 2.95%
Population-based descriptive study	Tokudome et al. (1989)	Japan	Entire residents of the Saga Prefecture (an endemic area in Japan)	Data from the Statistics Bureau in 1981	Data from a cancer registry in 1981–1983	Annual IR: (per 100,000 population aged 40–79 years)	NA
				M + F: 880,000	M: 36	M: 4.9∼12.6 (depend on age)	
					F: 33	F: 1.6∼8.1 (depend on age)	
			Estimated HTLV-1 carriers in the Saga Prefecture (an endemic area in Japan)	Data calculated by multiplying HTLV-1 positivity rate among blood donors with the number of the population in Saga	Data from a cancer registry in 1981–1983	Annual IR: (per 100,000 HTLV-1 carriers aged 40–79)	(40–79 years):
				M: 14,236	M: 36	M: 115.9	M: 4.5%
				F: 19,596	F: 33	F: 66.4	F: 2.6%
Nationwide hospital-based survey	Tajima (1990)	Japan	Whole Japanese population	Data from the Statistics Bureau in 1986	Data from 192 hospitals 1986–1987	Annual IR: (per 100,000 adults)	NA
				Total: 120,720,000	Total: 657	M: 4.04 (in Kyushu)	
				Kyushu: 14,460,000		F: 2.64 (in Kyushu)	
			Estimated HTLV-1 carriers in Japan	Data calculated by multiplying the HTLV-1 seropositivity rate in blood donors in an individual prefecture with the number of the population in this individual prefecture	Data from 192 hospitals 1986–1987	Annual IR (per 100,000 HTLV-1 carriers over 20 years old)	NA
				Total: 1,200,000	Total: 657	Total: 60	
Population-based descriptive study	Gérard et al. (1995)	French Guiana	Whole French Guiana population	Total 115,000	Enrolled in the study in 1990–1993	Crude annual IR (per 100,000 entire population) Total: 3.5	NA
					Total: 18	Crude annual IR in an endemic region (per 100,000 population) Total: 30
Cohort study (Miyazaki Cohort study)	Hisada et al. (1998a)	Japan	Residents in two HTLV-1 endemic villages in the Miyazaki Prefecture (an endemic area in Japan)	1,960 of whom 27% were HTLV-1 antibody-positive in 1984	Data in 1984–2000	NA	NA
	Okayama et al. (2004)			Total: 6		
Population-based descriptive study	Levine et al. (1999)	US	Central Brooklyn black community (an endemic area in New York)	Total: 1,184,670	Data from a survey in 1994	NA	NA
					M: 2		
					F:10		
Population-based descriptive study	Arisawa et al. (2000)	Japan	Entire residents of the Nagasaki Prefecture (an endemic area in Japan)	Data from the Statistics Bureau in 1990	Data from a cancer registry in 1985–1995	World age-standardized annual IR (cases/100,000 population):	NA
			M: 736,729	M: 567	M: 10.5		
				F: 826,230	F: 422	F: 6.0		
			Residents of 4 towns on the K Islands (a cluster regions in Nagasaki)	Data from the Statistics Bureau in 1990	Data from a cancer registry in 1985–1995	Crude IR (per 100,000 person-years of residents)	(30–79 years):
				M: 12,820	M: 24	M: 27.4	M: 1.7%
				F: 14,050	F: 16	F: 15.9	F: 0.7%
			HTLV-1 carriers of 4 towns on the K Islands (a cluster regions in Nagasaki)	Data from HTLV-1 screening in 1985–1996	Data from a cancer registry in 1985–1995	Crude IR (per 100,000 person-years of HTLV-1 carriers)	(30–79 years):
				M + F: 18,485	M: 24	M: 137.7	M: 6.6%
					F: 16	F: 57.4	F: 2.1%
Population-based descriptive study (NAACCR)	Yamamoto and Goodman (2008)	US	General population in US	Approximately 61% of the US population	Data from cancer registry in 1997–2002	Age adjusted to the 2000 US standard population per 100,000 population	NA
					M: 248	M: 0.05	
					F: 183	F: 0.03	
			White population in US	NA	M: 187	M: 0.05	NA
					F: 104	F: 0.02	
			Black population in US	NA	M: 46	M: 0.12	NA
					F: 69	F: 0.13	
Population-based descriptive study	Arisawa et al. (2009)	Japan	Entire residents of the Nagasaki Prefecture (an endemic area in Japan)	Data from the Statistics Bureau in 1995	Data from a cancer registry in 1985–2004	World age-standardized annual IR (per 100,000 population)	(30–99 years):
				M: 726,894	M: 1,022	M: 8.7	M: 0.88%
				F: 818,040	F: 829	F: 5.5	F: 0.57%
Hospital-based and Population-based descriptive study	Koga et al. (2010)	Japan	Estimated HTLV-1 carriers in Nagasaki City (an endemic area in Japan)	Data calculated by multiplying the HTLV-1 positivity rate in the University hospital with the number of the population census in Nagasaki City	Data from a cancer registry in 1990–2005	Annual IR (per 100,000 HTLV-1 carriers)	(30–79 years):
				M: 12,755	M: 188	M: 92	M: 7.29%
				F: 24,228	F: 172	F: 44	F: 3.78%
Nationwide hospital-based survey	Yamada et al. (2011)	Japan	Whole Japanese population	Data from the Statistics Bureau in 2006	Data from 156 hospitals 2006–2007	Annual IR (per 100,000 population)	NA
				Total: 127,053,000	Total: 910	Total: 0.91	
				Kyushu: 13,407,000	Kyusyu: 544	Kyushyu: 5.11	
			Estimated HTLV-1 carriers in Japan	Data calculated by multiplying the HTLV-1 seropositivity rate in blood donors in an individual prefecture by the number of the population in this individual prefecture	Data from 156 hospitals 2006–2007	Annual IR (per 100,000 HTLV-1 carriers over 20 years old)	M:8.73%
				Total: 1,078,722	Total: 910	Total: 106	F:5.14%

*NA, not available*.

Adult T-cell leukemia accounts for 51–59% of non-Hodgkin lymphoma (NHL) in HTLV-1 endemic areas in the Kyushu district, southwest Japan (Arisawa et al., 2000; Ohshima et al., 2002), which was extremely higher than that of nationwide data reporting that ATL accounts for 7.5% of all lymphomas (Lymphoma Study Group of Japanese Pathologists, 2000).

#### Annual mortality of ATL

**Figure 1:**
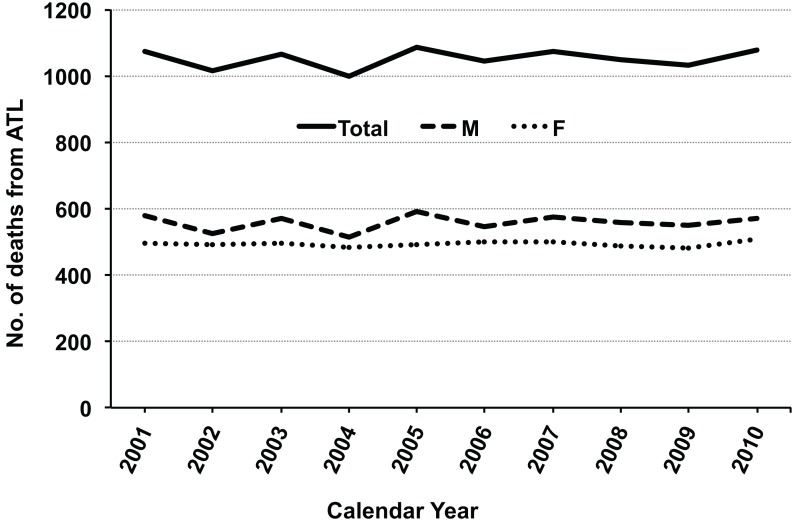
**Annual number of deaths from adult T-cell leukemia (ATL) during 2001–2010 in Japan**. The raw data were obtained from vital statistics in the Portal Site of Official Statistics of Japan (e-Stat; accessed April 8, 2012). Abbreviations: M, male; F, female.

Approximately 1,000 people die of ATL each year in Japan according to Japanese vital statistics data for 1998–2008 (Portal Site of Official Statistics of Japan, 2012; Figure 1). This indicates that infection with HTLV-1 was associated with approximately 1,000 deaths from ATL annually, with clustering in people aged over 50 years (Ikeda et al., 2012).

#### Annual incidence of ATL in nationwide studies

**Figure 2:**
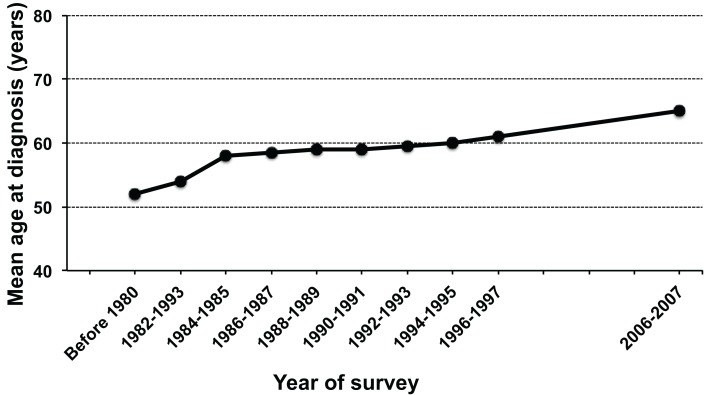
**Chronological changes in the mean age at diagnosis of adult T-cell leukemia (ATL) in Japan**. The figure was modified from Figure 2 in Yamada et al. (2011).

In the first nationwide hospital-based survey, 657 new cases of ATL were accumulated during 1986–1987, estimating the annual number of ATL in Japan to be approximately 700 cases (Tajima, 1990; Shimoyama, 1991). The new nationwide hospital-based survey was conducted recently, in which a total of 910 new cases of ATL were accumulated during 2006–2007, estimating the annual number of ATL in Japan to be approximately 1,000 cases (Yamada et al., 2011). In the new survey, two new findings were revealed in contrast to the first nationwide study. First, the age at diagnosis increased from a mean age of 52.7 years in the previous survey to 66.0 years in the new survey (Figure 2). Second, there were differences in the proportion of subtypes; the acute subtype accounted for the highest percentage (60.2%), followed by the lymphoma subtype (23.7%) in the previous survey, however, the percentage of the lymphoma subtype increased to 34.8%, contrary to the decrease in the acute subtype to be 46.7% in the new study. However, Takezaki et al. (1997) suggested that the annual incidence of ATL based on the nationwide hospital-based survey could be underestimated because approximately 65% of ATL cases might have been missed due to low response of the participating hospitals from endemic areas.

#### Annual incidence of ATL in HTLV-1 endemic areas

Results differ according to study methods and the HTLV-1 – positive rate of the study population. A series of cross-sectional survey for residents in Uwajima City (population size; 290, 464, HTLV-1-positive rate; 5.4% in men and 8.3% in women) reported that the annual incidence of ATL was estimated to be approximately 6.1 in adults aged over 30 years per 100,000 populations (Kondo et al., 1985, 1987, 1989). In another cross-sectional studies by the use of the regional cancer registry data in Nagasaki prefecture (an endemic area, the population size; 1.56 million), the age-standardized annual incidence rate of ATL (among 100,000 individuals aged 30 or older) was estimated to be 10.5 for men and 6.0 for women during 1985–1995 (Arisawa et al., 2000) and 8.7 for men and 5.5 for women during 1995–2004 (Arisawa et al., 2009). There was no significant decrease in the overall incidence rate between the two decades, however, age-specific incidence of ATL among those aged over 60 years increased significantly during 1995–2004 compared to the period of 1985–1995 (Arisawa et al., 2009).

#### Incidence of ATL among HTLV-1 carriers

In studies used blood donors seropositive for HTLV-1, the annual incidence of ATL was estimated to be approximately 60 per 100,000 HTLV-1 carriers over 20 years old in Japan (Tajima, 1990) or approximately 116 for men and 66 for women per 100,000 HTLV-1 carriers in Saga prefecture (an endemic area, the population size; 880,000; Tokudome et al., 1989). In a study used serological survey for residents in small cluster areas, The crude annual incidence of ATL was estimated to be 137.7 for men and 57.4 for women among 100,000 HTLV-1 carriers aged 30 years or older (Arisawa et al., 2000). Furthermore, in a study performed record linkage between the cancer registry and HTLV-1 carriers in hospital, the crude annual incidence of ATL was estimated to be 61 per 100,000 HTLV-1 carriers (Koga et al., 2010).

#### Lifetime risk of ATL among HTLV-1 carriers

For HTLV-1 carriers, the lifetime risk was estimated to be 4.5% for men and 2.6% for women in Saga prefecture (Tokudome et al., 1989), 6.6% for men and 2.1% for women in Nagasaki prefecture (Arisawa et al., 2000), 6.9% for men and 2.95% for women in Uwajima City (Kondo et al., 1989), and 7.29% for men and 3.78% for women in a hospital-based study (Koga et al., 2010).

In summary, in Japan, nearly 1,000 new cases of ATL are diagnosed and nearly 1,000 patients die of ATL each year over a period of 20 years. The annual incidence of ATL among HTLV-1 carriers is approximately 60 per 100,000 with the lifetime risk of 6–7% for men and 2–3% for women. The incidence was 1.35 times higher in men than in women, contrary to the higher HTLV-1-positive rate in women than in men. ATL occurs predominantly in elderly male carriers, and the mean age at diagnosis increased from the early 1950s in 1980 to the late 1960s recently. Most of Japanese epidemiological studies were population-based descriptive types using cancer registries, therefore those have limitations as follows; cases of smoldering ATL were excluded; hematological diagnoses were not performed. These limitations might have introduced an underestimation of the actual risk.

### East asia (excluding japan)

Although there were several reports of blood donor screening for HTLV-1, no epidemiological study of ATL has been published from East Asian countries other than Japan because of the lower prevalence of HTLV-1 (less than 0.1%). Nevertheless, several case series of ATL were available. The first case of ATL was reported in Taiwan in 1985 (Chen et al., 1985), in Korea in 1987 (Lee et al., 1987), and in China in 1995 (Zhuo et al., 1995). In Hong Kong, since the first case of ATL was reported in 1994 (Liang, 1994), all patients with T-cell lymphoma have been routinely screened for HTLV-1 antibody. In a registration study of lymphoma between 1993 and 2002 in Hong Kong, six cases of ATL were diagnosed among 5,911 lymphomas, in which ATL contributed to 0.1% of all cases of lymphoma and 1.3% of T-cell lymphoma (Au and Lo, 2005). Recently, 17 cases of ATL were reported from Taiwan (Lee et al., 2010), of those approximately 40% of the patients co-infected with HBV and HCV, which may be a characteristic of the Taiwanese ATL.

### Middle east

The prevalence of HTLV-1 infection among healthy subjects is reported to be very low, less than 0.1%, in Lebanon, Saudi Arabia, Egypt, and Kuwait (Proietti et al., 2005). However, there are some areas with a very high rate of HTLV-1 infection.

Northeast province of Iran (Mashhad, Sabzevar, and Neyshabour) and Urmia are known to be an endemic area for HTLV-1, where the prevalence of HTLV-1 infection was reported to be 0.34–0.77% in blood donors (Abbaszadegan et al., 2003; Khameneh et al., 2008), 1.7–12% in cross-sectional studies (Meytes et al., 1990; Safai et al., 1996; Hedayati-Moghaddam et al., 2011; Azarpazhooh et al., 2012), and 2–3% in community-based population (Rafatpanah et al., 2011).

Romania is also suggested to be an endemic area for HTLV-1 because antibodies to HTLV-1 were found in 0.64% of blood donors (Paun et al., 1994), which was an extremely higher seroprevalence rate than in Europe and the USA. In Israel, HTLV-1 seropositive were discovered only in 0.0018% out of 276,000 blood donations, but a very high rate of infection (over 20%) has been identified among a segregated community of Jews originated from the city of Mashhad in Iran (Miller et al., 1998).

Although, there are several clinical studies for ATL patients in the Middle East (Kchour et al., 2007, 2009), epidemiological studies regarding incidence and prevalence of ATL were not available in literature from the Middle East. There were several case reports of ATL, most of which were Mashhad origins or Romanian origins (Sidi et al., 1990; Veelken et al., 1996; Shtalrid et al., 2005; Bitar et al., 2009).

### United states

HTLV-1 and ATL are extremely rare in North America. Several ATL cases have been reported sporadically (Catovsky et al., 1982). Most of the cases were migrants from endemic areas. A population-based survey reported that the annual incidence in African Americans in central Brooklyn (population size; 1,184,670) was estimated to be approximately 3.2 per 100,000 person-years (Levine et al., 1999). An interesting finding in their study was that the male-to-female ratio of 1:3 was different from the male dominance reported in Japan. Recent cancer registry systems for hematological malignancies allow a precise evaluation of epidemiological features of ATL in the USA. In a recent report from the North American Association of Central Cancer Registries (NAACCR; Yamamoto and Goodman, 2008), a total of 431 cases (248 men and 183 women) of ATL (ICD-O-3 code; 9,827) were registered between 1997–2002, showing that the age adjusted incidence rate was 0.05 for men and 0.03 for women per 100,000 population. The study also reported a racial difference in the incidence rate, showing that African Americans had the highest rates of ATL (0.12 for men and 0.13 for women per 100,000 population). A possible explanation for this observation might be the higher number of migrants from endemic areas of the Caribbean and parts of Sub-Saharan Africa rather than a racial difference in susceptibility.

### The caribbean

In the early 1980s, eight patients were diagnosed with ATL in the USA, and all of them were Blacks from the Caribbean (Blattner et al., 1982). Since then, Central/South America and the Caribbean are known as areas of high prevalence of HTLV-1. Although there is no concrete epidemiological report regarding the incidence or prevalence of ATL from Central and South America, several case series have been published. A regional registration study of Jamaica reported a total of 126 cases of ATL (acute 46.8%, lymphoma 27%, chronic 20.6%, and smoldering 5.6%) between January 1985 and July 1995 (Hanchard, 1996). The mean age was 43 years old (17–85 years old), which is similar to that reported in Brazil (43 years; Pombo de Oliveira et al., 1995) but younger than that in Japan (50–60 years; Yamaguchi et al., 1987). There is definite evidence that the age at diagnosis in Central/South America and the Caribbean is younger than that in Japan. This difference in the age at diagnosis might be due to different environmental backgrounds.

### Central and south america

In Central and South America, HTLV-I has been shown to be endemic mainly in populations of African ancestry and in some populations of Japanese origin.

Brazil has the highest HTLV-1 seroprevalence rate in healthy subjects (approximately 1%), especially in Rio de Janeiro and Salvador (1.8%) on the northeast coast of the country where the population is largely of African descent. ATL accounts for approximately 30% of patients with T-cell malignancies in Brazil (Pombo de Oliveira et al., 1995; Farias de Carvalho et al., 1997). A Brazilian ATLL Study Group identified 195 cases of ATL in the national registry of T-cell malignancies between 1994 and 1998 (Pombo de Oliveira et al., 1999), but no epidemiological indicators were available. In Argentina, HTLV-1 infection is known to be highly prevalent among Native Americans living in the Andes, and ATL accounts for approximately 14.7% of patients with lymphoid malignancies (Marin et al., 2002).

Chile is a non-tropical country but small case series of ATL patients have been reported frequently (Cabrera et al., 1994, 1999, 2003). The characteristics of Chilean ATL were reported that the most of patients were of Caucasian origin, and age at diagnosis (50 years old) was younger than Japanese patients but older than those from other Latin American countries. According to the recent pathological study in Chile, ATL accounts for 0.5% of patients with of NHL (Cabrera et al., 2012).

French Guiana (population 115,000), an overseas French administrative district located on the northeast coast of the South American continent between Brazil and Surinam, is also known to be an area of high endemicity for HTLV-I (Plancoulaine et al., 1998; Talarmin et al., 1999, Pouliquen et al., 2004). Although the population consists of various ethnic groups, a high seroprevalence of HTLV-I (8%) and a high incidence of cases of ATL were found among the Noirs-Marrons, an isolated population descended from Surinam slaves (Gérard et al., 1995; Tuppin et al., 1995; Plancoulaine et al., 1998). An epidemiological study was performed in French Guiana to determine the prevalence and incidence of ATL (Gérard et al., 1995). Only 18 patients with ATL (8 acute forms, 8 lymphoma types, and 2 smoldering cases) were enrolled during 1990–1993 and the annual crude incidence rate was estimated to be around 3.5 per 100,000 populations. However, in a small remote ethnic group of African origin (around 6200 inhabitants), the annual crude incidence rate was the highest to be around 30 per 100,000 populations.

### Africa and europe

In Africa, a high HTLV-I seroprevalence rate (>2% in the adult population) has been reported in sub-Saharan African countries, especially in Gabon (Hunsmann et al., 1984; Delaporte et al., 1988; Gessain, 1996; Etenna et al., 2008; Gonçalves et al., 2010). Although there are many reports regarding the HTLV-I seroprevalence rates in African countries, only a few epidemiological studies of ATL were available. In a case-control study including NHL and control that performed in Gabon, only four cases of the 26 patients with NHL fitted the criteria of ATL (Delaporte et al., 1993), but further information on epidemiological feature of ATL was not available.

In Europe, HTLV-1 is endemic in Southern Italy (Manzari et al., 1985). Several case series of ATL were reported from Europe (Manzari et al., 1985; Gessain et al., 1990). Most of ATL patients were African origin from high-HTLV-1-endemic areas (West Indies, Nigeria, and other African areas); however, some patients had no background regarding endemic areas (Manzari et al., 1985).

## Risk Factors for ATL in HTLV-1 Carriers

Although a variety of genetic abnormalities due to HTLV-1 infection have been reported to explain the characteristics of ATL oncogenesis, HTLV-1 infection alone is not sufficient to develop ATL from HTLV-1 carrier status. Risk factors for developing ATL in HTLV-1 carriers have been investigated in many epidemiological and clinical studies (Table 2).

**Table 2:** **Risk factors for the development of ATL with regard to the HTLV-1 carrier status**.

	Reference
**Host susceptibility**
Vertical infection with HTLV-1 as infant	Murphy et al. (1989)
Attained at an age of >50 years	Many references
Male sex	Many references
HLA-A*26, HLA-B*4002, HLA-B*4006, and HLA-B*4801 (Japanese ATL)	Yashiki et al. (2001)
Co-infected with *Strongyloides stercoralis*	
**Laboratory markers**
A high level of sil-2R, more than 500 U/ml	Arisaw et al. (2002)
A high level of anti-HTLV-1, titer more than × 1,024	Arisaw et al. (2002)
A high level of circulating abnormal lymphocytes, more than 0.6%	Hisada et al. (1998a)
A low level of of anti-Tax reactivity	Hisada et al. (1998b)
A high level of white blood cell count, more than 9,000/μL	Imaizumi et al. (2005)
**Viral markers**
A higher HTLV-1 proviral load level, more than 4 copies per 100 PBMCs	Iwanaga et al. (2010)

*ATL, adult T-cell leukemia; HTLV-1, human T-cell leukemia virus type 1; HLA, human leukocyte antigen; PBMC, peripheral blood mononuclear cell; sIL-2R, soluble interleukin-2 receptor*.

### Host susceptibility

Age is a well-known risk factor for the development of ATL. ATL occurs mostly in adults, at least 20–30 years after HTLV-1 infection. However, the age at onset differs across geographic areas, which may be affected by racial or environmental characteristics. In Japan in the early 1980s, an average age at diagnosis of ATL was reported to be individuals in their early 1950s (The T- and B-Cell Malignancy Study Group, 1981, 1985), but the age at diagnosis increased yearly, reaching 65 years in the latest nationwide survey for ATL (Yamada et al., 2011). However, the average age at diagnosis of ATL in Jamaican and Brazilian series was reported to be individuals in the 1940s (43 years in Jamaica and 44 years in Brazil; Hanchard, 1996; Pombo de Oliveira et al., 1999), which is younger than that in Japan (Yamaguchi et al., 1987).

The age at the time of HTLV-1 infection is also a very important risk factor for the development of ATL. Individuals infected in childhood (vertical transmission) may be at higher risk for developing ATL (Murphy et al., 1989). ATL seldom develops in individuals infected in adulthood, although no epidemiological study has proven this fact. There was one case report describing that a female HTLV-1 carrier known as conclusively transmitted horizontally by her partner developed ATL (Sakuma et al., 1988). To clarify whether or not ATL develops among individuals infected in adulthood, a large prospective follow-up study is required.

Male sex is considered a risk factor for ATL. In most studies from Japan, the incidence of ATL is two- and threefold higher in male carriers than in female carriers, which is contrary to the higher rate of HTLV-1 positivity in women than in men. However, a population-based survey in central Brooklyn reported that the annual incidence of ATL was higher in women than in men (male-to-female ratio of 1:3; Levine et al., 1999). Modeling data from Jamaican series also showed a higher cumulative lifetime risk of ATL in women than in men (4.0% for men and 4.2% for women; Murphy et al., 1989). The reason for the sex-related differences in the incidence rate of ATL between Japan and other regions is unknown.

It seems unlikely that there are apparent ethnical differences in susceptibility to infection by HTLV-1 and developing ATL. A higher incidence of ATL was found individual of African origin than in others (Manzari et al., 1985; Gessain et al., 1990; Yamamoto and Goodman, 2008), however, most of patients of African origin came from HTLV-1 endemic areas.

Earlier epidemiologic studies have found that ATL patients are more likely to have a family history of lymphoid malignancy (Ichimaru et al., 1979; The T- and B-Cell Malignancy Study Group, 1981). Since then, several host genetic background factors influencing the onset of ATL have been investigated. Human leukocyte antigen (HLA) is a candidate for the genetic factors controlling the immune response against the viral antigen. Specific HLA antigen alleles have been reported to be associated with an increased risk of developing ATL (Uno et al., 1988). The allele frequencies of HLA-A*26, HLA-B*4002, HLA-B*4006, and HLA-B*4801 were significantly higher in ATL patients than in asymptomatic HTLV-1 carriers in southern Japan, and ATL patients possessing these alleles developed ATL 12.6 years earlier than patients with other alleles (Yashiki et al., 2001). Ethnic differences in HLA alleles related to ATL were also investigated in another study (Sonoda et al., 2011).

HTLV-1 carriers with abnormal immune system may be at high-risk of developing ATL. Several studies reported that HTLV-1 carriers co-infected with *Strongyloides stercoralis* are considered a high-risk group for developing ATL because of the clonal proliferation of HTLV-1-infected lymphocytes and high proviral load (Nakada et al., 1987; Yamaguchi et al., 1988; Plumelle et al., 1997; Gabet et al., 2000). Satoh et al. (2002) suggested that *S. stercoralis* infection induces polyclonal expansion of HTLV-1-infected cells by activating the interleukin 2/interleukin 2 receptor (IL-2/IL-2R) system in dually infected carriers, which may be a precipitating factor for ATL. The immunosuppressive state has been reported to potentially contribute to ATL development in HTLV-1 carriers. There were several case reports of ATL developed in HTLV-1 carriers undergoing immunosuppressive treatment after living-donor liver transplantation (Kawano et al., 2006; Yoshizumi et al., 2012) and kidney transplantation (Hoshida et al., 2001).

### Laboratory markers

Several laboratory abnormalities were found to be markers for the development of ATL. Kamihira et al. (1994) measured prospectively soluble IL-2R (sIL-2R) levels and lactate dehydrogenase (LDH) levels in HTLV-1 carriers, reporting that the increasing level of sIL-2R may be a more sensitive indicator of ATL than LDH. A nested case-control study also showed that high levels of sIL-2R (more than 500 U/mL) and HTLV-1 antibody titers (more than 1,024) were independently associated with an increased risk of developing ATL (Arisaw et al., 2002). Imaizumi et al. (2005) analyzed the outcomes of 50 HTLV-1 carriers with monoclonal proliferation of HTLV-1-infected T cells in a 20-year follow-up study, reporting that a high white blood cell count more than 9,000/μL was a potential prognostic factor for developing ATL, even after adjustment for age, sex, and relative lymphocyte counts.

A series of the Miyazaki Cohort Study (population size; 1,960 people, of whom 27% were HTLV-1 antibody-positive) reported that an HTLV-1 carrier with a high anti-HTLV-1 titer (odds ratio; 1.6), a high number of circulating abnormal lymphocytes, and a low anti-Tax reactivity were associated with a greater risk of developing ATL (Mueller et al., 1996; Hisada et al., 1998a,b). Recently, an international ATL Cohort Consortium study by merging eight cohorts from Japan, Jamaica, the United States, and Brazil examined serologic markers of HTLV-I pathogenesis and host immunity in 53 ATL cases and 150 matched asymptomatic HTLV-I carriers (Birmann et al., 2011). The study confirmed that above-median sIL-2R and anti-Tax seropositivity were independently associated with an increased ATL risk, and found that above-median total immunoglobulin E levels predicted a lower ATL risk.

Aberrant expression of cell-surface antigens is usually used for clinical routine diagnosis on ATL. ATL cells phenotypically express CD4, CCR4, and CD25. However, data of cell-surface antigens rarely used for a prognostic marker of ATL from HTLV-I carriers. Two studies reported that expression of CD3, CD7, and CD26 on HTLV-1-infected cells were diminished in acute and chronic ATL and those were slightly down-regulated in smoldering ATL (Tsuji et al., 2004; Tian et al., 2011). These results suggest that the down-regulation of those cell-surface antigens could be possible predict markers for the early phase leukemogenesis of ATL from HTLV-1 carriers. A resent study serially evaluated cell-surface antigens on HTLV-1-infected cells in HTLV-1 carriers, smoldering ATL, and chronic ATL, by taking into consideration the pattern of Southern blot hybridization and proviral load (Kamihira et al., 2012). The report suggests that the decreasing expression of CD26 and the decreasing ratio of CD26/CD25 are novel biomarkers for prediction of clonal bands and discrimination of carriers and smoldering ATL.

### Provirus-integration status

Among HTLV-1 carriers, there exist a group of cases having the monoclonal integration of HTLV-1 proviral DNA in mononuclear cells without signs of malignant proliferation or clinical signs and symptoms related to leukemia (Ikeda et al., 1993). Such carriers have been suggested to be a high-risk group of developing ATL, but their prognosis varied from being stable carriers for long to developing ATL (Ikeda et al., 1993; Imaizumi et al., 2005). There are only a few epidemiological studies to investigate the significance of the provirus-integration status on non-malignant infected cells from asymptomatic HTLV-1 carriers.

Nakada et al. (1987) reported that patients with *S. stercoralis* infection and co-infected with HTLV-1 had a high frequency (35%) of patients presenting a monoclonal integration of HTLV-1 proviral DNA in their blood lymphocytes. Carvalho and Da Fonseca Porto (2004) also The author also found a correlation between monoclonal integration of proviral DNA and abnormal lymphocytes in peripheral blood, with a trend for greater severity of the parasitic infection. Although several studies reported that HTLV-1 carriers co-infected with *S. stercoralis* are considered a high-risk group for developing ATL (Nakada et al., 1987; Yamaguchi et al., 1988; Plumelle et al., 1997; Gabet et al., 2000), no study investigated the clinical significance of the monoclonal integration of HTLV-1 proviral DNA in their blood lymphocytes in HTLV-1 carriers with *S. stercoralis*.

### Proviral load

In the area of viral oncogenesis, there are accumulated data indicating a relationship between an increased viral load and viral-associated malignancies. HTLV-1 proviral DNA load in the peripheral blood mononuclear cells (PBMCs) are also evaluated in some epidemiological and clinical studies to support the hypothesis that increased HTLV-1 proviral load level is an important predictor of developing ATL.

A cross-sectional study (Manns et al., 1999) and a series of the Miyazaki cohort study (Tachibana et al., 1992; Hisada et al., 1998a,b; Okayama et al., 2004) reported that HTLV-1 proviral load level was higher in HTLV-1 carriers who developed ATL than in asymptomatic HTLV-1 carriers. However, the proviral load was measured only in a small number of subjects in the above literature.

Several large-scale prospective studies support results from the previous small studies that an increased HTLV-1 proviral load is an important predictor of developing ATL. In Japan in 2002, a nationwide prospective cohort study for asymptomatic HTLV-1 carriers, the Joint Study on Predisposing Factors of ATL Development (JSPFAD), was initiated (Yamaguchi et al., 2007) to investigate viral- and host-specific determinants of the development of ATL in more detail. In the cohort of 1,218 asymptomatic HTLV-1 carriers (426 men and 792 women), 14 subjects progressed to overt ATL during a follow-up of 1981.2 person-years (Iwanaga et al., 2010). All of the 14 subjects were among those with the highest group of baseline proviral load (range, 4.17–28.58 copies/100 PBMCs). Multivariate Cox analyses indicated that a higher proviral load (more than 4 copies/100 PBMCs) is an independent risk factor for progression of ATL, even after adjusting for sex, age, family history of ATL, and other possible risk factors. The result indicated that HTLV-1 carriers with higher HTLV-1 proviral load levels belong to the high-risk group of carriers who develop ATL and in whom any measures to prevent the development of ATL should be instituted.

Nevertheless, the association between HTLV-1 proviral load and disease development remains unclear because a higher proviral load is also an important predictor in patients with HTLV-1-associated myelopathy/tropical spastic paraparesis (HAM/TSP). Further viral markers are needed to determine the function of a higher HTLV-1 proviral load to direct the way to developing ATL or developing HAM/TSP from HTLV-1 carriers.

## Concluding Remarks

Although many prior studies found important epidemiological evidence on ATL and risk factors for the development of ATL in HTLV-1 carriers, limited data are available on the valid annual incidence of ATL from longitudinal prospective studies. Existing predisposing factors are still insufficient to explain the characteristics of ATL oncogenesis. Unknown risk factors may be involved in the acquisition of malignant characteristics of HTLV-1 infected cells. Further well-designed epidemiological studies are needed to fully understand the oncogenesis of ATL.

Even though the incidence of ATL is relatively low among HTLV-1 carriers and a novel promising agent, mogamulizumab (humanized anti-CCR4 monoclonal antibody), is released (Ishida et al., 2003, 2012), preventing new HTLV-1 infections and the development of ATL are major public health concerns in HTLV-1 endemic countries in the world. In Japan, there are approximately one million of HTLV-1 carriers, 1,000 new ATL cases, and 1,000 new deaths from ATL every year. However, only recently has the Japanese government for the first time begun to implement a nationwide comprehensive package of measures covering the prevention of mother-to-child HTLV-1 transmission and the development of medical researches on HTLV-1 and ATL (http://www.kantei.go.jp/foreign/kan/actions/201009/13htlv_e.html). The challenge in the next few years will be to reduce the number of HTLV-1 carriers, to develop an easy method that allows identification of high-risk carriers, and to implement earlier therapeutic interventions for carriers with high-risk markers.

## Conflict of Interest Statement

The authors declare that the research was conducted in the absence of any commercial or financial relationships that could be construed as a potential conflict of interest.
